# Heart rate variability as a measure of mental stress in surgery: a systematic review

**DOI:** 10.1007/s00420-020-01525-6

**Published:** 2020-03-25

**Authors:** Anne-Fleur The, Iris Reijmerink, Maarten van der Laan, Fokie Cnossen

**Affiliations:** 1 Division of Vascular Surgery, Department of Surgery, University Medical Center Groningen, Groningen, The Netherlands; 2 Department of Artificial Intelligence, Bernoulli Institute of Mathematics, Computer Science and Artificial Intelligence, Faculty of Science and Engineering, University of Groningen, Nijenborgh 4, 9747 AG Groningen, The Netherlands

**Keywords:** Heart rate variability, Mental stress, Surgery, Occupational stress

## Abstract

**Purpose:**

There is increasing interest in the use of heart rate variability (HRV) as an objective measurement of mental stress in the surgical setting. To identify areas of improvement, the aim of our study was to review current use of HRV measurements in the surgical setting, evaluate the different methods used for the analysis of HRV, and to assess whether HRV is being measured correctly.

**Methods:**

A systematic review was performed according to the Preferred Reporting Items for Systematic reviews and Meta-Analyses (PRISMA). 17 studies regarding HRV as a measurement of mental stress in the surgical setting were included and analysed.

**Results:**

24% of the studies performed long-term measurements (24 h and longer) to assess the long-term effects of and recovery from mental stress. In 24% of the studies, artefact correction took place.

**Conclusions:**

HRV showed to be a good objective assessment method of stress induced in the workplace environment: it was able to pinpoint stressors during operations, determine which operating techniques induced most stress for surgeons, and indicate differences in stress levels between performing and assisting surgery. For future research, this review recommends using singular guidelines to standardize research, and performing artefact correction. This will improve further evaluation of the long-term effects of mental stress and its recovery.

**Electronic supplementary material:**

The online version of this article (10.1007/s00420-020-01525-6) contains supplementary material, which is available to authorized users.

## Introduction

Surgery is one of the most demanding safety–critical professions. The operating theatre can be a stressful environment (Menon et al. 2016; Demirtas et al. 2004). There is ample evidence that when physicians are under stress, quality of care is indeed reduced (Wallace et al. 2009). Stress also affects the physicians themselves. Long-term exposure to stress has been associated with a number of ill-health outcomes such as burn-out (Unterbrink et al 2007), cardiovascular diseases (Peter and Siegrist 2000) and depression (Oskrochi et al. 2018). Detection of mental stress is therefore not only extremely important to detect, reduce and prevent the adverse effects of mental stress on quality of care, but also on the physicians themselves.

While accurate and reliable measurements of stress are important, measuring stress is challenging, as stress is perceived and coped with differently by individuals. Measures of stress vary from questionnaires to biochemical evaluations such as cortisol measurements to heart rate variability. There is an increasing interest into more objective measurements of mental stress, as these cannot easily be manipulated and provide an accurate representation of the stress level (Amirian et al. 2014).

One objective measurement which can be used for measuring mental stress in the surgical setting is heart rate variability (HRV) (Jarvalen-Pasasen et al. 2018; Thielmann and Böckelmann 2016). Heart rate variability is the variation in the interval between successive normal NN intervals, which has been shown to decrease as mental stress increases. Variations in heart rate (HRV) can be calculated in the time domain and in the frequency domain [as a power spectral density (PSD) analysis] as well as with non-linear analysis (Sassi et al. 2015; Sammito et al. 2015). In both time and frequency domain analyses, the time intervals between successive normal NN intervals are determined first. The NN intervals are recorded by measuring the difference between two R waves in the QRS complex. Time domain indices of HRV are more direct measures of variations in interbeat intervals (IBI) and include *SDNN* (standard deviation of IBI), *SDANN* (the standard deviation of the average IBI), *RMSSD* (the square root of the mean squared differences of successive IBIs), and *NN50* (the number of interval differences of successive IBIs larger than 50 ms). While some specific time domain indices are thought to reflect parasympathetic control of cardiac output (with cardiac output rising in response to stress), other time domain indices cannot be assigned clearly (Schaffer et al. 2017). Time domain indices do not provide detailed information on sympathetic control; the main advantage of using time domain measures is that they are easy to calculate. Frequency domain measures perform more complex calculations on IBI (Fourier transforms), expressing variability in terms of a power density spectrum (energy in specific frequency bands). Frequency domain measures can be calculated for any frequency band, but the most common ones are *LF* (low frequency, 0.04–0.15 Hz) and *HF* (high frequency, 0.15–0.4 Hz), but also *VLF* (very low frequency, < 0.04 Hz) is sometimes used, as is the *LF/HF ratio* (ratio low frequency/high frequency). Specific frequency bands are thought to reflect sympathetic and/or parasympathetic control, and therefore give more detailed information on the effects of stress on the autonomic nervous system.

However, calculating time and frequency domain measures of HRV is not straightforward and a number of factors need to be considered before analysis. For example, artefact correction is essential. HRV analysis should always be performed on normal-to-normal beat interval data (i.e. all intervals between adjacent R waves in the QRS complexes resulting from sinus node depolarizations) (Lippman et al. 1994). Artefacts such as missed, extra or misaligned beats can significantly alter HRV parameters (Peltola et al. 2012), and analyses using sports watches without correcting the raw data, for example, deliver unreliable results (Sammito and Böckelmann 2016).

Non-linear dynamics methods indicate qualitative aspects of the series of NN intervals (Sammito et al. 2015). These methods can be used for both long-term and short-term measures and has the advantage of being less prone to artefacts.

When evaluating HR and HRV in the field of occupational medicine, several modifiable and non-modifiable factors should be taken in account, as they can affect HR and HRV, the most relevant being alcohol, breathing, fitness activities, sex, cardiovascular diseases, temperature, body weight, noise, age, psychiatric disorders, smoking, hazardous substances, shift work including night shift, metabolic disorders, stress/mental tension and circadian rhythm/time of the day (Sammito et al. 2015).

The aim of this review was to evaluate the current use of HRV measurements within the surgical setting: with what purpose are they used, how long is it measured; to assess which methods are being used for analysing HRV (time domain/frequency domain/non-linear dynamics); and to assess whether HRV was measured correctly (i.e. whether artefacts were corrected).

## Methods

### Search strategy and study eligibility

This review was conducted and reported according to the Preferred Reporting items for Systematic reviews and Meta-Analyses (PRISMA) statement. The databases Medline, Embase, and PsycINFO were searched up to June 19, 2018 for studies regarding heart rate variability as a measurement of mental stress in the surgical setting. The search strategy was created in collaboration with a clinical librarian (see Appendix 1). For the database searches, Medical Subject Heading terms and additional free entry terms for stress, heart rate variability and terms related to the surgical profession were used. Duplicates were removed. Title and abstract of all studies were screened by the authors. The reference lists of the included articles were screened for additional relevant publications.

**Fig. 1:**
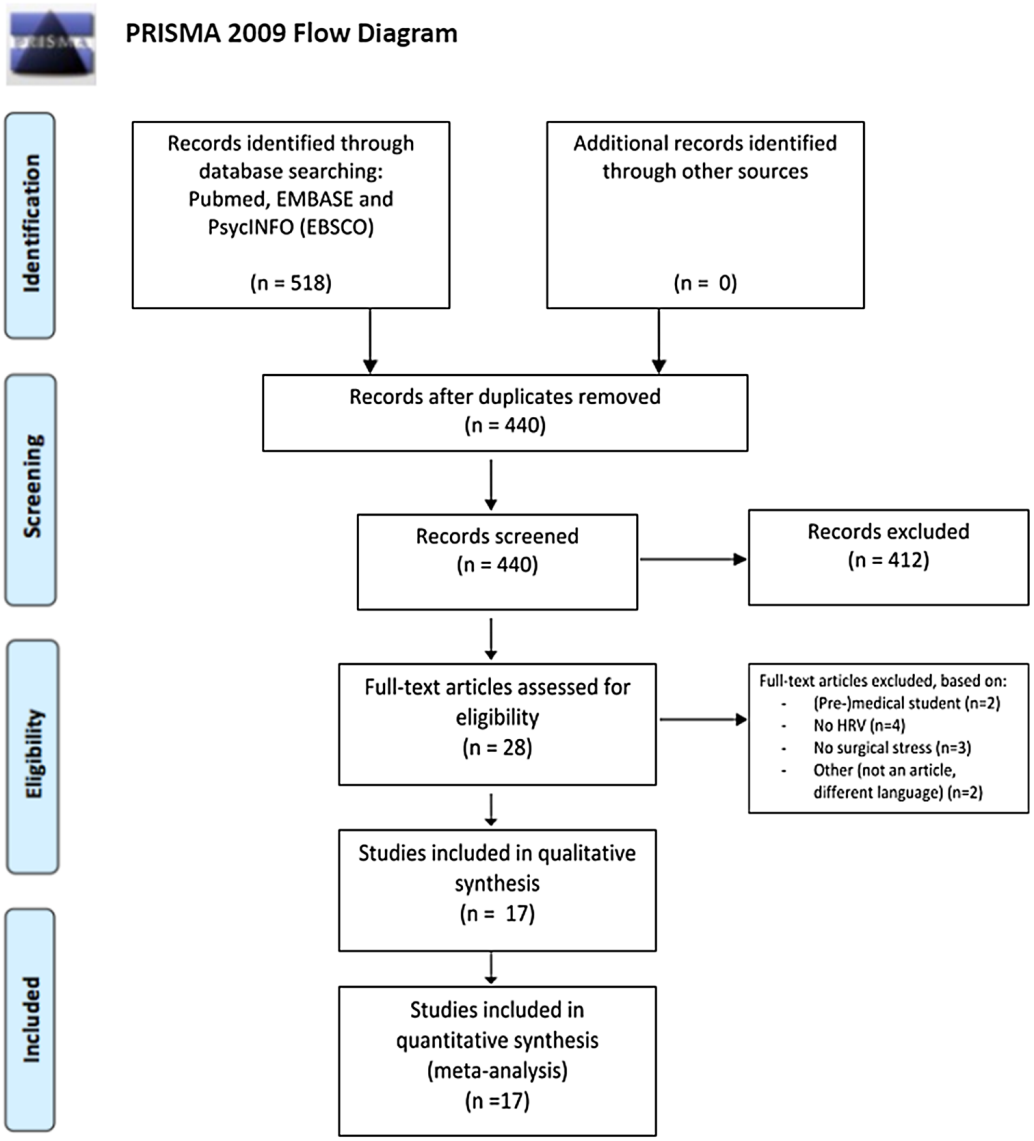
PRISMA flowchart

Studies were selected for full text analysis based on a predetermined set of inclusion and exclusion criteria. Studies that were included described a surgical procedure affected by mental stress, which was measured by means of HRV. Both studies with surgeons as well as with surgical residents as the subject of the study were included. Articles based on physical stress, non-surgical professions, medical students, no HRV parameters and no surgical outcome were excluded from this analysis. The study inclusion process is summarized in a PRISMA flowchart (Fig. 1). Differences in inclusion were resolved by plenary discussion. Studies were screened for full text if dubiety for inclusion was present amongst the authors. A total of 17 studies met the inclusion criteria and were thus included. A summary of the selected studies is presented in Table 1.

**Table 1:** Evidence table

References	Study design	*N*	Assessment of mental stress
Amirian et al. (2014)	Prospective cohort study	29	HRV
Bohm et al. (2001)	Prospective randomized study	2	HRV, HR
Demirtas et al. (2004)	Prospective cohort study	12	HRV
Ganne et al. (2016)	Prospective cohort study	4	HRV, HR
Heemskerk et al. (2014)	Prospective randomized study	2	HRV, HR
Jones et al. (2015)	Prospective cohort study	6	HRV, STAI
Joseph et al. (2016)	Prospective observational study	19	HRV, STAI, NASA task load index
Klein et al. (2010)	Prospective case–control	10	HRV, VAS
Langelotz et al. (2008)	Prospective cohort study	8	HRV, HR, VAS
Malmberg et al. (2011)	Prospective cohort study	35	HRV
Prichard et al. (2012)	Prospective cohort study	2	HRV, HR
Rieger et al. (2014)	Cross-sectional study	20	HRV, HR, STAI
Song et al. (2009)	Prospective cohort study	1	HRV
Weenk et al. (2018)	Explorative study	20	HRV, short version STAI
Wetzel et al. (2011)	Randomized, controlled, intervention study	16	HRV, HR, STAI, observer rating by surgical assistant, C-HRVf, salivary cortisol
Wetzel et al. (2010)	Prospective cohort study	20	HRV, HR, STAI, observer rating by surgical assistant, C-HRV, salivary cortisol
Yamanouchi et al. (2015)	Prospective cohort study	2	HRV

### Data extraction and quality assessment

Data were extracted from the eligible articles by all investigators. Discrepancies were immediately resolved by plenary discussion. The following data were extracted from each article: number of participants; aim of study; type of stress measurements; HRV measurement devices; HRV parameters (time and frequency domain); artefact corrections; factors possibly interfering with HRV; length of HRV measurements; additional measurements used for assessment of mental stress and main findings. The methodological quality of the studies included was assessed using the Newcastle–Ottawa Scale, which assessed the selection of study groups, the comparability of study groups and the ascertainment of either the exposure or outcome.

### Statistical analysis

As a result of the large heterogeneity of the included studies, it was not possible to perform a meta-analysis. Data were therefore summarized and displayed in descriptive statistics.

## Results

A total of 518 articles derived from Pubmed, EMBASE and PsycINFO were identified. 78 duplicates were removed, and thus 440 articles were screened for eligibility. 412 articles were excluded based on title and abstract. 11 articles were excluded based on full-text analysis. These articles included medical students as participants (*n* = 2), no HRV measurement present (*n* = 4), no surgical stress measurement (*n* = 2) or other reasons (*n* = 3). A total of 17 studies were included in the systematic review.

All included studies describe a surgical setting in which the surgeon's mental stress is measured by means of HRV. 8 of the 17 included studies had less than nine participants included in their studies.

### HRV parameters

53% of the studies (*n* = 9) evaluated HRV by both domain measures and 35% (*n* = 6) of studies evaluated HRV only by frequency domain measures, while 6% of studies (*n* = 1) evaluated HRV solely by time domain measures. Finally, 6% of studies (*n* = 1) used a different method of evaluating HRV, namely beat-to-beat HRV compared with baseline HRV.

In 88% of the studies (*n* = 15), frequency domain measures were used to evaluate HRV. In all of these studies, a low-frequency (LF) component of 0.04–0.15 Hz and a high-frequency (HF) component of 0.15–0.4 Hz were calculated/determined. In 82% of the studies (*n* = 14), the LF/HF ratio was calculated based on these components, the remaining 18% of the studies (*n* = 3) did not calculate the LF/HF ratio. In 29% of the studies (*n* = 5), an additional very-low frequency (VLF) component of < 0.04 Hz was calculated as well as the HF and LF components. Furthermore, 18% of the studies (*n* = 3) included the total power (TP), the sum of all frequency components, in their analysis. 6% of the studies (*n* = 1) evaluated HRV by means of HFnu, the high-frequency component in normalized units (HFnu = ((HF/TP-VLF)) × 100).

In 59% of the studies (*n* = 10), time domain measures were used to evaluate HRV. Multiple time domain measures can be evaluated. A variety of time measures can be found in a singular study, and thus overlap between time domain measures can be present.

18% of the studies (*n* = 3) evaluated the mean R–R interval, which is the mean time elapsed between successive heartbeats. In 41% of the studies (*n* = 7) SDNN, the standard deviation of normal to normal interval was calculated. In 35% of the studies (*n* = 6), RMSSD, the square root of the mean normal to normal interval, was calculated. 18% of studies calculated pNN50, which is the percentage of adjacent pairs of normal to normal intervals differing by more than 50 ms in the recordings. 12% of the studies (*n* = 2) included the HRV coefficient (C_HRV), which was calculated by the following formula: C_HRV = SDNN/NN × 100. Finally, 6% of the studies (*n* = 1) calculated the difference between the longest and shortest R–R interval.

### Artefact correction

For accurate HRV measures, a correction of artefacts needs to be performed (Lippman et al. 1994). Artefacts such as missed, extra or misaligned beats can cause significant alterations into HRV parameters, and therefore any aberrant beat should be corrected prior to HRV analysis (Peltola et al. 2012). This systematic review therefore analysed whether the included studies included artefact correction. 24% (*n* = 4) of the studies performed artefact correction in their analysis. If artefact correction took place, recordings were visually inspected and manually corrected.

### HRV measurement purpose

The included studies were classified into subgroups according to why the study used HRV measures of mental stress: (1) studies evaluating whether mental stress was present in certain situations (*n* = 9; for results, see Table 2), (2) studies evaluating the differences in mental stress between different operating techniques or operating room environments (*n* = 3; for results, see Table 3), (3) studies evaluating the changes in mental stress between performing surgery and assisting surgery (*n* = 3; for results, see Table 4), and (4) remaining studies not classifiable to the other subgroups (*n* = 3; for results, see Table 5). One study compared mental stress between different operating techniques as well as between performing and assisting surgery, so fits in both (2) and (3).

**Table 2:** Overview of aim, studies evaluating whether mental stress was present in certain situations

References	No. of participants	Aim	Measures of stress	Measurement procedure	Main findings
Demirtas et al. (2004)	12 surgeons [5 operators (plastic surgery staff) and 7 junior residents acting as assistants]	To assess the mental burden of surgeons, dedicated to operative stress, by utilizing HRV indices	HRV, HR	Assistants monitored for 2 days, operators 4 days (from 8:00 am to 6:00 pm). Half recordings were operating days, other half were office days (baseline)	Surgeons: increase HR, LF, LF/HF ratio, decrease HF during rhinoplasty operations compared to baseline Assistant: increase LF, LF/HF ratio, decrease HF during operations. Sympathetic arousal of operators was more pronounced than that of assistants
Jones et al. (2015)	Six consultant colorectal surgeons	To evaluate surgical stress in the clinical setting using HRV in combination with a validated subjective assessment tool	HRV, State Trait Anxiety Inventory (STAI) short version	Baseline STAI and HRV were recorded at 08:00 on the day of surgery. Further HRV recordings were taken at predetermined operative steps. STAI score was obtained immediately after each operation	Increase LF/HF ratio from baseline to mean operative recordings. In 75% of operations classified as stressful procedures based on STAI. Univariate correlation analysis of STAI and mean operative LF/HF showed a significant, positive correlation Mesorectal dissection was reported as the most stressful step in 75% of operations
Langelotz et al. (2008)	Eight surgical residents and specialists	To determine the specific effects of working long hours in surgery and potential cardiac stress in the individual surgeon by measuring HRV	HRV; HR; visual analogue scale (VAS) on stress and fatigue	HRV was measured during a resting period at the beginning of the 24 h shift, after 12 h, and at the end of the shift. The shift consisted of a workday of 8.5 h + 15.5 h of on-call service. Before each recording, participants assessed their fatigue and stress levels on a VAS of 0–100. Total amount of rest during the shift was recorded. Measurements were repeated over 10 24-h shifts	VAS scores for fatigue were higher after 12 and 24 h than at the beginning of the day, and correlated with the amount of work hours during the 24-h shift. Lower HR before shift vs after, no correlation with stress/fatigue scores. SDNN, RMSSD, and pNN50, increased over 24 h. HF + LF increased, LF/HF ratio remained unchanged because of the rise in parallel. Correlations of perceived stress during and after the shift with HRV parameters were found, but no such correlations were present for fatigue
Malmberg et al. (2011)	Two groups: 19 anaesthesiologists (ANEST) and 16 paediatricians/ENT surgeons (PENT)	To investigate whether HRV differed during recovery from day work and night-call duty between distinct physician specialities	HRV, mean HR	Holter ECG was made on three occasions: (1) from one ordinary workday to the next (16:00–16:00), (2) during night-call duty (16:00–08:00), and (3) continuously during the following post-call period (08:00–08:00). Also measured blind during “unwinding” (21:00–22:00)	ANEST: lower HF, HFnu. HF lower in the evening after daytime work and when on night call, but not in the evening post-night call, when compared with PENT. Every one HFnu lower post-daytime work and when on night call compared with post-call. Thus, the physiological recovery after night duty seemed sufficient in terms of HRV patterns for HFnu. However, the less dynamic HRV after daytime work and during night-call duty in the ANEST group may indicate a higher value
Rieger et al. (2014)	Six residents, five fellows, five attending, and four chiefs of medicine	To examine the specific effects of intraoperative stress on the cardiovascular system by measuring HR and HRV	HR; HRV; STAI	Measurement of HRV took place during the whole work day and a resting period at night (24 h total). Baseline values were assessed from nighttime recording. Based on their perceived stress (STAI), surgeons were classified as stressed or non-stressed	7 physicians felt intraoperatively stressed, whereas 12 did not. 1 did not fill in STAI postoperatively. Only differences in HRV at night were found. LF, VLF, and TP of non-stressed surgeons were significantly higher than those of stressed surgeons. Higher HR in OR for both stressed and non-stressed surgeons. Higher RR interval of non-stressed at night compared to stressed. Measurements in both groups. Non-stressed participants showed significant differences in relative changes of total power and SDNN, whereas stressed physicians did not
Yamanouchi et al. (2015)	Two surgeons, performing five PD and four LDLT	To evaluate mental stress of surgeons before, during and after operations, especially during pancreaticoduodenectomy (PD) and living donor liver transplantation (LDLT)	HRV	The two surgeons wore the device from 1 h before operation to 1 h after operation. The device monitored data every minute	In PD: lower HF and higher LF/HF during operation, than before the operation, and did not return to the baseline level 1 h after the operation In LDLT, HF was decreased in two and the LF/HF increased in three cases during operation vs before the operation. In all, HF was decreased and/or LF/HF increased during the reconstruction of the vessels or bile ducts than during the removal of the liver
Ganne et al. (2016)	Four neurosurgeons	To evaluate HRV of the neurosurgeons during microsurgical clipping of aneurysm by using continuous real time monitoring of the ECG intraoperatively	HRV; HR	All surgeries were performed during the daytime between 9 AM and 5 PM. A continuous recording of the ECG was done throughout the procedure from skin incision to haemostasis	Increase in HR and decrease in power values in all the frequency bands from baseline up to clipping. Tended to return to the baseline during haemostasis. LF/HF ratio increased from baseline to haemostasis. Progressive reduction in RMSSD, as the average HR increased from baseline to clipping. Reversal of these changes was noticed from clipping stage to haemostasis stage. The maximum HR was noted around the perianeurysmal dissection stage and clipping with the lowest HRV during clipping. There was tachycardia and a reduction in the R–R interval variation at the time of clipping
Joseph et al. (2016)	19 surgeons (7 junior residents, 7 senior residents, 8 attending surgeons)	To assess the level of stress during trauma activation and emergency surgery using subjective data and objective HRV	HRV, STAI, NASA task load index	Monitor was worn for whole 24 h on call. Before start, members were asked to sit for a duration of 5 min to record the baseline HR. Single investigator followed the trauma team to log events, such as operation time	Stress level increased during trauma activations and operations regardless of the level of training. The attending surgeons had significantly lower stress when compared with senior residents and junior residents during trauma activation and emergency surgery. The level of stress was similar between junior residents and senior residents during trauma activation and emergency surgery
Weenk et al. (2018)	Five consultants, seven fellows and senior residents, and eight junior residents	To identify activities and risk factors of stress in surgeons and residents using a novel patch sensor (The	HRV; short STAI; HR	Baseline patch data and STAI score were collected during 15 min total rest. Next, data were collected for the next 48–72 h. STAI was filled out before and after each surgical procedure. Log book was kept with type and time of daily activities and also physical activity	Decrease SDNN, decrease RMSSD, increase LF/HF ratio and 3 × increase stress percentage during surgery vs. baseline. Lower SDNN and RMSSD and higher stress percentage during surgery vs. non-surgical activities. Fellows and senior residents higher stress percentages and lower SDNN and RMSSD than consultants during surgery. Lower RMSSD scores in junior residents. Significant difference between baseline STAI scores and preoperative STAI scores.15/42 surgical procedures with complete STAI identified as stressful. No difference in SDNN, RMSSD, LF/HF ratio and stress percentage between stressful and non-stressful procedures

**Table 3:** Overview of aim, studies evaluating the changes in mental stress between different operating techniques or operating room environments

References	No. participants	Aim	Measures of stress	Measurement procedure	Main findings
Böhm et al. (2001)	Two surgeons (1 more experienced (> 80 laparoscopic colectomies) and one less experienced (20 laparoscopic colectomies)	To investigate whether surgeons experience more signs of mental strain during laparoscopic vs conventional sigmoid resection	HRV, HR	Two surgeons performed ten conventional and ten laparoscopic sigmoid resections, alternating roles as primary surgeon and assistant. ECG was run continuously throughout the procedure	HF was lower, LF was higher, and the LF/HF ratio was much higher in laparoscopic surgery
Klein et al. (2010)	Ten experienced surgeons (individual experience > 200 laparoscopic cholecystectomies)	To examine whether optimized ergonomics and technical aids within a modern OR affect psychological and physiological stress in experienced laparoscopic surgeons	HRV; visual analogue scale on time pressure, effort, imaginable performance, frustration, satisfaction, degree of pain	HRV was measured throughout the procedure: initial and last 5 min were excluded. Parameters measuring physical strain and pain were recorded immediately before and after surgery	The physical strain and pain of the surgeon was lower in a modern OR compared with a standard OR. No changes in HRV were present, and thus no significant differences in the perceived psychological stress of the surgeon
Heemskerk et al. (2014)	Two experienced surgeons	To investigate the level of mental strain experienced by the surgeon performing robot-assisted laparoscopic surgery compared to conventional laparoscopic surgery	HRV, HR	Using one baseline and six well-defined stages in the surgical procedure (laparoscopic cholecystectomy), seven interval tachograms of 5 min beginning at the start of each stage were selected and analysed	RC took longer to perform than CC. Baseline is equal for both groups, but in the course of the operation, CC leads to a higher mean HR compared to baseline, whereas RC leads to a lower HR. When looking at LF/HF ratio, baseline is similar for both groups, but during the operation, CC leads to a significant higher LF/HF ratio than RC

**Table 4:** Overview of aim and studies evaluating the changes in mental stress between performing surgery and assisting surgery

Study	No. of participants	Aim	Measures of stress	Measurement procedure	Main finding
Böhm et al. (2001)	Two surgeons (1 more experienced (> 80 laparoscopic colectomies) and one less experienced (20 laparoscopic colectomies)	To investigate whether surgeons experience more signs of mental strain when performing vs assisting surgery	HRV, HR	Two surgeons performed ten conventional and ten laparoscopic sigmoid resections, alternating roles as primary surgeon and assistant. ECG was run continuously throughout the procedure	While the HR and LF/HF ratio of the surgeon was much higher, the assistant was much more relaxed (higher HF). The experienced surgeon was more relaxed than the less experienced (lower LF/HF ratio despite higher overall HR). The experience of the assistant was not found to influence HRV
Song et al. (2009)	One attending-consultant surgeon	To determine whether there are differences in HRV when performing vs supervising and assisting CABG surgery	HRV	One surgeon performed 30 CABG surgeries and assisted 20 CABG surgeries. ECG was run continuously from the moment the surgeon walked into the OR to the moment the surgeon finished operation. CABG was divided into six steps	As surgeon: LF/HF ratio highest in the beginning of all operations, stabilized thereafter, and decreased towards the end. As assistant: LF/HF ratio highest in the phase of heart arrest and coronary anastomosis
Prichard et al. (2012)	Two consultant surgeons and three surgical endocrine fellows	To determine whether instructing surgical trainees in technically demanding procedures causes alterations in HRV and mental strain in supervising surgeons	HRV; HR	The consultant group performed 50 lobectomies as primary operator, and 50 as surgical assistant/teacher; similar for fellow group. Within each total thyroidectomy the consultants performed one lobectomy and the fellows the other. ECG was run throughout total thyroidectomy	Surgical fellows: no difference in HR determined by surgical role. Energy consumption higher with primary operator. No difference in SDNN between roles. Higher LF/HF ratio with primary operators. Consultant surgeons: no difference in HR determined by surgical role. No difference in energy consumption. Higher SDNN and RMSSD when acting as the primary operators. Decrease in HF with surgical teachers. Increase in LF/HF ratio when attending surgeons were teaching the fellows

**Table 5:** Overview of aim; remaining studies not classifiable to the other subgroups

Study	No. of participants	Aim	Measures of stress	Measurement procedure	Main findings
Wetzel et al. (2010)	30 surgeons (21 surgical residents, 9 attendings surgeons); 13 low experience (2–8 years’ experience), 17 high experience (10–34 years’ experience)	To investigate the effects of surgeons’ stress levels and coping strategies on surgical performance during simulated operations	STAI, observer rating by surgical assistant, HR, HRV, salivary cortisol	Procedure followed a standardized protocol of two simulated CEAs: the first was non-crisis scenario, in second multiple crisis. HR and HRV were measured continuously throughout both procedures. Stress questionnaires were completed after each simulation, an interview with the surgeon was conducted and saliva was obtained	During the non-crisis simulation, a high coping score and experience significantly enhanced the end product. During the crisis simulation, a significant beneficial effect of the interaction of high experience and low stress on all performance measures was found. Coping significantly enhanced nontechnical skills
Wetzel et al. (2011)	16 surgical residents who were able to perform a CEA as the primary surgeon	To investigate the effects of the stress management training (SMT) on surgeons’ operative performance during a simulated carotid endarterectomy (CEA)	Short version STAI; observer rating by surgical assistant (scale 0–10), HR; HRV; salivary cortisol	Two groups of eight participants each performed two crisis CEA simulations. The intervention group received the SMT after performing simulation 1. The control group received no treatment No. of surgical coping strategies, surgical performance and stress was measured. HRV was measured throughout the procedure	The intervention group and the control group did not differ in baseline levels. In the intervention group during the second simulation: higher number of coping strategies, higher C_HRV, increased nontechnical skills, lower observed stress and salivary cortisol, higher technical skills and quality surgical end product. In the control group, there were no significant changes
Amirian et al. (2014)	29 surgeons (interns, residents, attending surgeons)	To clarify the effect of a 17-h night shift on surgeons’ HRV	HR, HRV	Surgeons were monitored for 48 h (8 am morning precall, continued through night shift 3.30 pm—8.30 am, till 8 am morning post-call). Surgeons were monitored for psychomotor performance, cognition, circadian rhythm, sleep and fatigue	HR was decreased precall vs on call. Increased HF precall vs on call. LF/HF ratio lower precall vs on call. No correlation between LF/HF ratio and performance in laparoscopic simulation (performance = time in laparoscopic simulator sessions). No post-call HRV monitoring was performed

### Duration of HRV measurements

The duration of HRV measurements differed between studies. This is a reflection of the fact that different studies evaluated different procedures and different participants and had different aims. In this systematic review, studies were divided into three groups: long duration (24 h and longer), short duration (less than 24 h ranging from 11 min to 16 h), and studies measuring throughout the whole procedure (which did not mention the exact duration of the HRV measurements). 24% of the studies (*n* = 4) were long duration, 53% (*n* = 9) were short duration and 24% (*n* = 4) were whole procedures.

### Factors affecting HRV

Certain factors such as smoking, alcohol consumption, caffeine consumption, medication use and the presence of cardiovascular diseases or diabetes are known to affect HRV.

In 76% of the studies (*n* = 13) included at least one of these factors was mentioned in the method. 41% of the studies (*n* = 7) assessed smoking habits of the participants; of those, only non-smokers were included in four studies; in two studies, some participants smoked on a regular basis; and in one study participants were asked not to smoke for 24 h before the measurement. 18% of the studies (*n* = 3) assessed alcohol consumption among the participants: in two studies participants were asked not to consume alcohol 24 h before the procedure, and one study reported that all participants had a low to moderate general alcohol consumption.

18% of the studies (*n* = 3) assessed caffeine consumption: in one study, participants were asked not to consume caffeine 24 h before the procedure, in one study participants were asked not to consume caffeine on the day of the procedure, and in one study there were no constrictions regarding caffeine consumption.

65% of the studies (*n* = 11) assessed medication use amongst participants: nine studies reported no use of any medication, one study reported no use of beta-blockers, and one study reported looking into medication use, but no outcome was mentioned in the article.

53% of the studies (*n* = 9) assessed the presence of cardiovascular disease among participants: eight studies reported no presence of disease, and one study reported no family history of cardiac diseases amongst all participants. Finally, 18% of the studies (*n* = 3) assessed the presence of diabetes among participants: all three of these studies reported the absence of diabetes among all participants.

All information concerning the measurement of heart rate variability and factors affecting heart rate variability is summarized in Table 6.

**Table 6:** Overview of HRV parameters, artefact corrections and other information in the included studies

References	HRV device	HRV parameters		Artefact correction	Other	Confounding factors: smoking (S), alcohol (A), caffeine (C), medication (M), cardiovascular disease (CD), and diabetes (D) in participants?
		Time domain	Frequency domain			
Demirtas et al. (2004)	Three-lead digital ambulatory Holter recorder (Lifecard CF Digital Compact Flash Card Recorder)	N/A	HF (0.15–0.4 Hz); LF (0.04–0.15 Hz); LF/HF ratio	Yes, manual corrections by blind physician using an editor program	Data sample rate: 1024/sec	S: no, 20% operators smoke, 43% of assistants M, CD: no A, C, D: N/A
Jones et al. (2015)	Wireless Polar RS800CX monitor	N/A	HF (0.15–0.4 Hz); LF (0.04–0.15 Hz); VLF (< 0.04 Hz); LF/HF ratio	N/A	N/A	S: all non-smokers, C, M, CD: no A, D: N/A
Böhm et al. (2001)	Solid-state minimized autonomous recording device (brand not mentioned)	Mean R-R interval; SDNN; difference longest and shortest R–R interval	HF (0.15–0.4 Hz); LF (0.04–0.15 Hz); LF/HF ratio	Yes, visual checks and manual corrections	Data sample rate: 400/s	S: non-smokers, A, C, M, CD, D: N/A
Langelotz et al. (2008)	Polar S810 Heart Rate Monitor (Polar Electro Inc., Lake Success, New York)	SDNN; RMSSD; pNN50	HF (0.15–0.4 Hz); LF (0.04–0.15 Hz); LF/HF ratio	N/A	N/A	M, CD, D: no S, A, C: N/A
Song et al. (2009)	Solid-state very small autonomous recording device (RAC-3103, Nihon Kohden, Japan)	N/A	HF (0.15–0.4 Hz); LF (0.04–0.15 Hz); LF/HF ratio	N/A	N/A	All N/A
Wetzel et al. (2010)	Wireless HR monitor (S801i, Polar, Kempele, Finland)	R–R interval; SDNN; C_HRV	LF/HF ratio	N/A	N/A	All N/A
Klein et al. (2010)	MEDILOG AR12 recorder (Oxford Instruments, Tubney Woods, Abingdon, Oxfordshire, UK)	SDNN; RMSSD;	HF (0.15–0.4 Hz); LF (0.04–0.15 Hz); LF/HF- ratio	N/A	N/A	S: all non-smokers M: No A, C, CD, D: N/A
Malmberg et al. (2011)	Digital, portable monitoring unit for Holter ECG (DXP 1000; Braemar systems, Chicago, IL, USA)	N/A	HF (0.15–0.4 Hz); LF (0.04–0.15 Hz); VLF (< 0.04 Hz); TP; HFnu; LF/HF ratio	N/A	Sampling frequency: 125 Hz	S: 1 ANEST was a smoker A: all low—moderate general alcohol consumption CD, D: No C, M: N/A
Wetzel et al. (2011)	Wireless heart rate monitor (S801i, Polar, Kempele, Finland)	C_HRV	N/A	N/A	N/A	All N/A
Prichard et al. (2012)	Polar RS 800 heart rate Monitor (Polar Electro, Inc., Lake Success, NY)	SDNN; RMSSD; pNN50	HF (0.15–0.4 Hz); LF (0.04–0.15 Hz) LF/HF ratio	N/A	N/A	S: all non-smokers C: forbidden for 1 h preoperatively M, CD, D: no A: N/A
Rieger et al. (2014)	Equivital sensor system EQ-01 (Hidalgo Ltd., Cambridge)	SDNN; RMSSD; pNN50;	HF (0.15–0.4 Hz); LF (0.04–0.15 Hz); VLF (< 0.04 Hz); TP	N/A	N/A	M, CD: no S, A, C, D: N/A
Amirian et al. (2014)	Medilog AR12 recorder (Oxford Instruments Tubney Woods) with a three-channel, five-lead recording	N/A	HF (0.15–0.4 Hz); LF (0.04–0.15 Hz); LF/HF ratio	Yes, recordings were manually viewed and excluded for noise, ectopy and missing beats, and only intervals with > 90% valid data were included		N/A
Heemskerk et al. (2014)	Standard bipolar electrocardiogram (ECG)	Mean R–R intervals;	HF (0.15–0.4 Hz); LF (0.04–0.15 Hz); VLF (< 0.04 Hz); LF/HF- ratio	N/A		Sample rate of 400/s
Yamanouch i et al. (2015)	Small monitoring device (brand not mentioned)	N/A	HF (0.15–0.4 Hz); LF (0.04–0.15 Hz); LF/HF ratio	N/A		N/A
Ganne et al. (2016)	Bioharness (Zephyr Technologies, Annapolis, MD), a telemetric ECG recording system	RMSSD	HF (0.15–0.4 Hz); LF (0.04–0.15 Hz)’ LF/HF ratio; TP	Yes: data were visually inspected for artefacts, discontinuous signal, excess noise and ectopics. R peaks were determined by identifying the maximum value above a threshold		Sample rate: 1024 Hz
Joseph et al. (2016)	Zephyr’s BioHarness 3.0 (Zephyr Technology, Annapolis, MD)	High-level mental strain = beat-to-beat HRV < 60% baseline HRV Low-level mental strain = beat-to-beat HRV 60–85% baseline HRV No mental strain = beat-to-beat HRV > 85% baseline HRV		N/A		N/A
Weenk et al. (2018)	The HealthPatch, a flexible self- adhesive patch containing two ECG electrodes and a battery	SDNN; RMSSD	HF (0.15–0.4 Hz); LF (0.04–0.15 Hz); VLF (< 0.04 Hz); LF/HF ratio	Yes/no: technical failures and side effects of the HealthPatch were documented		Low sample frequency of the patch, causing possible inaccuracy in LF and HF data

### Additional measurements used for the assessment of mental stress

In almost all included studies, HRV was not the only used measurement of mental stress. Only 18% of the studies (*n* = 3) used HRV as the only measurement of mental stress. 35% of the studies (*n* = 6) used heart rate (HR) in combination with HRV to measure mental stress. 12% (*n* = 2) used the STAI (State Trait Anxiety Inventory) in addition to HRV. The remaining 35% of the studies (*n* = 6) used a combination of different subjective and objective measures of stress and fatigue. Combinations included HR and STAI (*n* = 1), HR and VAS (visual analogue scale) (*n* = 2), NASA-TLX (NASA Task Load Index) and STAI (*n* = 1) and STAI in combination with observer ratings, HR, HR and salivary cortisol (*n* = 2).

## Discussion

This systematic review evaluated the different methods used in the studies for the analysis of HRV (time domain/frequency domain/non-linear dynamics), to assess whether HRV is being measured correctly (i.e. whether artefacts were corrected) and to evaluate the current use of HRV measurements in the surgical setting (short-term vs. long-term measurements) to identify areas for improvement in future HRV research within the surgical setting.

This systematic review showed that HRV shows to be a good objective assessment method of stress induced in the surgical setting: it was able to pinpoint stressors during operations, determine which operating techniques induced most stress for surgeons, and indicate differences in stress levels between performing and assisting surgery. In addition, this review showed a lack of artefact correction: even though artefact correction is essential for reliable HRV calculations, only four studies (24%, *n* = 4) mentioned correcting for artefacts. The review also showed studies evaluating the long-term effects of mental stress and its recovery were lacking.

Almost all studies in this review used frequency domain measures, while half of the studies also included time domain measures. The fact that frequency domain measures are being used more often might be because of the fact that when analysing stationary short-term recordings, the task force recommends the use of frequency domain methods (Task Force of the European Society of Cardiology and the North American Society of Pacing and Electrophysiology 1996). The third method that can be used for calculating variations in heart rate are non-linear analyses, but these methods were not used in any of the included studies. In theory, this is a third method of cardiologists that can be used in HRV research; however because of the characterizing complex systems, successful application in the medical science fields is restricted For future research, the standards of measurement, physiological interpretation, and clinical use can be used to standardize the research into HRV as a measure of stress.

When evaluating the studies included, different goals can be identified for the use of HRV. These goals can be measuring stress during a specific operation, or assessing changes in stress levels between various surgical environments. HRV showed to be a good objective assessment method of stress induced in the workplace environment and was able to pinpoint stressors during operations. In addition, HRV was able to determine which operating techniques provided most stress for surgeons and to determine differences in stress levels between performing and assisting in the surgical procedure. Although different purposes for using HRV were found, the majority of studies had the same overall interest: measuring stress at a specific moment in time, namely during an operation. The included studies were mainly focused on the evaluation of short-term stress, instead of long-term stress and its recovery as the majority of the studies had a short duration of measurement.

Although short-duration measurements can inform us of the level of mental stress during the time frame or situation of interest, measurements of longer duration provide us with vital information on the recovery of stress. Long-term measurements (24 h or more), as opposed to short-term HRV monitoring, enable assessing stress and recovery patterns during normal working hours as well as during leisure time and during sleep (Jarvelin-Pasanen et al. 2018).

Only four of the included studies performed long-term measurements and investigated the long-term effects and recovery of mental stress. These studies found that working night shifts decreased the HRV of surgeons (Amirian et al. 2014) and that higher perceived stress in the operating room is associated with a decreased HRV at night (Rieger et al. 2014). This seems to indicate that stress increases during night shifts and that surgeons are still recovering from high stress of the operating room at night. To identify the long-term effects of stress and prevent its adverse effects on surgeons’ health, more research is needed with long-term HRV measurements, also to better understand if and how surgeons recover from mental stress during working hours.

As heart rate variability is a measure with complex underlying physiological mechanisms, it can be affected by many confounding factors, such as age, weight, physical activity, cardiac innervation, cigarette smoking, alcohol consumption, caffeine consumption, medication use, and core temperature (Jarvelin-Pasanen et al. 2018). The majority of the included studies reported on some of the above-mentioned factors and used these factors as exclusion criteria. Because of the interpersonal differences in HRV, it is recommended participants always serve as their own control.

Heart rate variability is an objective and reliable way of non-invasively monitoring stress in the clinical situation (Böhm et al. 2001; Prichard et al. 2012; Song et al. 2009). This review shows that HRV can be used successfully for different purposes to assess mental stress in the surgical setting, including the effect of operating techniques/environment on mental stress of surgeons and the change in mental stress between performing surgery and assisting. In addition, HRV shows to be a good objective assessment method of stress induced in the workplace environment, as it is able to pinpoint stressors during operations. This review also showed that the current studies are mainly focussed on the short-term measurement of mental stress. There is thus a lack of studies on the long-term effects of mental stress on surgeons, and its recovery.

To standardize HRV research, we further recommend that future research adheres to a single guideline, with using artefact correction to be the most pressing issue.

## Electronic supplementary material

Below is the link to the electronic supplementary material.

Supplementary file1 (DOCX 12 kb)

## Abbreviations

HRV: Heart rate variability
PRISMA: Preferred Reporting Items for Systematic reviews and Meta-Analyses
PSD: Power spectral density
IBI: Interbeat intervals
SDNN: Standard deviation of IBI
SDANN: Standard deviation of the average IBI
RMSSD: Square root of the mean squared differences of successive IBIs
NN50: Number of interval differences of successive IBIs larger than 50 ms
LF: Low frequency, 0.04–0.15 Hz
HF: High frequency, 0.15–0.4 Hz
VLF: Very low frequency
LF/HF ratio: Ratio of low frequency/high frequency
Normal-to-normal beat interval data: All intervals between adjacent R waves in the QRS complexes resulting from sinus node depolarizations
TP: Total power
HFnu: High-frequency component in normalized units
C_HRV: HRV coefficient
HR: Heart rate
STAI: State Trait Anxiety Inventory
VAS: Visual analogue scale
NASA-TLX: NASA task load index
N: Number of participants
pNN50: Percentage of adjacent pairs of normal to normal intervals differing by more than 50 ms in the recording.
RC: Robot-assisted cholecystectomy
CC: Conventional cholecystectomy
ECG: Electrocardiogram
OR: Operating room
CABG: Coronary artery bypass grafting
SMT: Stress management training
CEA: Carotid endarterectomy
