# Supplementary material for: Heart rate variability as a measure of mental stress in surgery: a systematic review
Source: Int Arch Occup Environ Health. 2020 Mar 25;93(7):805–21. doi: 10.1007/s00420-020-01525-6 (PMC7452878; doi:10.1007/s00420-020-01525-6)
Supplement: Supplementary file 1 — Supplementary file1 (DOCX 12 kb) [file 420_2020_1525_MOESM1_ESM.docx]

**Supplement 1: Search String Clinical Librarian**

**Pubmed**

("Surgeons"[Mesh] OR surgeon*[tiab] OR neurosurgeon*[tiab] OR surgical perform*[tiab] OR operator stress[tiab] OR intraoperative stress[tiab] OR intra-operative stress[tiab] OR surgical skill*[tiab] OR surgical pract*[tiab] OR surgical resident*[tiab])

AND

(heart rate varia*[tiab] OR "Heart Rate"[Mesh] OR heart rate[ti] OR hrv[tiab])

265 hits (19-6-2018)

**EMBASE**

('surgeon'/exp OR (surgeon* OR neurosurgeon* OR ‘surgical perform*’ OR ‘operator stress’ OR ‘intraoperative stress’ OR ‘intra-operative stress’ OR ‘surgical skill*’ OR ‘surgical pract*’ OR ‘surgical resident*’):ab,ti)
AND

('heart rate variability'/exp OR ‘heart rate’/mj OR ('heart rate'/de AND ('mental stress'/exp OR 'clinical competence'/exp)) OR (‘heart rate’ NEAR/3 variab*):ab,ti OR hrv:ab,ti OR ‘heart rate’:ti)

166 titels (19-6-2018)

**PsycINFO (EBSCO)**(DE "Surgeons" OR (surgeon* OR neurosurgeon* OR “surgical perform*” OR “operator stress” OR “intraoperative stress” OR “intra-operative stress” OR “surgical skill*” OR “surgical pract*” OR “surgical resident*”))

AND

(DE "Heart Rate" OR (“heart rate” N3 variab*) OR “hrv” OR TI “heart rate”)

87 titles
